# Notices, Reviews, Etc.

**Published:** 1860-01

**Authors:** 


### Notices, Reviews, Etc.

*American Medical Gazette*, New-York, monthly, $2 a year. Edited by L. Meredith Reese, LL.D., 10 Union square.

*Boston Medical and Surgical Journal*, monthly, $3 a year. 8vo, in its 51st vol.

*College Journal*, $2 a year, Cincinnati, Ohio.

*Medico-Chirurgical Review*, quarterly, $3 a year. Republished from London by the Messrs. Wood, 389 Broadway, New-York. Standard publication.

*Eclectic Medical Journal*, Philadelphia, Pa., monthly, $2 a year. William Paine, M.D., editor and publisher.

*Scalpel*, New-York. Edited by Edward L. Dixon, 42 Fifth avenue, New-York. Quarterly, $1 a year, 8vo.

*Massachusetts Teacher*, Boston, Charles Ansorge, editor, $1 a year.

*Merry's Museum*, New-York, for boys and girls, $1 a year.

*The Hesperian*, monthly, San Francisco, Cal. Edited by Mrs. F. H. Day.

*Blackwood's Magazine*, monthly, $3 a year. Republished promptly by Leonard Scott & Co., 79 Fulton street, New-York. Also

*North British Review*, quarterly, $3 a year. Republished by the same house.

*The London Quarterly Review*, $3 a year. *The Edinburgh Review*, $3 a year.

*The Westminster Review*, $3 a year.

These four *Reviews* and *Blackwood's Magazine*, whose contributors are among the finest minds and the ablest writers of Great Britain, are afforded for $10.

The *Christian Review*, $3 a year, quarterly, by Sheldon & Co., 115 Nassau street, New-York, is the organ of the Baptist Church, and is now in its 25th vol.

*The Pacific Expositor*, San Francisco, Cal., monthly, $3 a year. Edited by Rev. W. A. Scott, D.D.

*The Presbyterian Expositor*, $2.00 a year a year, Chicago, Ill. Edited by Rev. N. L. Rice, D.D., Professor in the North-Western Theological Seminary, and is devoted to the exposition of the doctrines of the Presbyterian Church.

*The Presbytery Reporter*, Chicago, $1 a year, monthly.

*Presbyterian Magazine*, $1 a year. Published monthly at Philadelphia, Rev. Dr. Van Rensselaer, editor.

*American Agriculturist*, $1 a year. Issued monthly by Orange Judd, A.M. Published also in German.

*American Phrenological Journal*, monthly, quarto, $1 a year. By Fowler & Wells, New-York.

*Water-Cure Journal*. Same size, price, and publishers.

*Life Illustrated*, same publishers, weekly, $1 a year. No. 308 Broadway.

*Scientific American*, weekly, $2 a year. Published by Munn & Co., 37 Park Row, New-York. The best publication of its kind in the world.

*Home Journal*, $2 a year, weekly, 107 Fulton st., New-York. By Morris & Willis.

*Musical World*, weekly, $2 a year. R. Storrs Willis, editor, 379 Broadway. In its 22d volume.

*Mothers' Journal*, $1 a year, monthly, New-York. Edited by Mrs. Hiscox.

*New-York Teacher*, $1 a year, Albany, N. Y.

*Home Monthly*, Buffalo, N. Y., $1.50 a year. Mrs. Airey & Gildersleeve, Eds.

*Western Farmer*, Chicago, Ill., $1 a year.

*Home and School Journal*, Chicago, Ill., $1 a year.

*The Farmers' Monthly*, 8vo, Detroit, Mich., $1 a year.

*American Farmer*, Baltimore, monthly, $2 a year.

*Eclectic Med. Jour.*, $2 a year, Philadelphia.

*Millennial Harbinger*, $1 a year, monthly A. Campbell, editor, Bethany, Va.

*Evangelical Repository*, Philadelphia, Pa , $1 a year, monthly.

*Godey's Lady's Book*, $3 a year, Philadelphia. The queen of pictorial monthlies.

*Ladies' Home Magazine*, Philadelphia, $2 a year. Useful and safe for all.

*The Home*, Boston, $2 a year. A monthly for the family ; always instructive.

